# Probe set filtering increases correlation between Affymetrix GeneChip and qRT-PCR expression measurements

**DOI:** 10.1186/1471-2105-11-104

**Published:** 2010-02-24

**Authors:** Jakub Mieczkowski, Magdalena E Tyburczy, Michal Dabrowski, Piotr Pokarowski

**Affiliations:** 1 Laboratory of Transcription Regulation, Department of Cell Biology, The Nencki Institute of Experimental Biology, Pasteur 3, 02-093 Warsaw, Poland; 2 Institute of Informatics, Faculty of Mathematics, Informatics and Mechanics, Banacha 2, University of Warsaw, 02-097 Warsaw, Poland

## Abstract

**Background:**

Affymetrix GeneChip microarrays are popular platforms for expression profiling in two types of studies: detection of differential expression computed by p-values of *t*-test and estimation of fold change between analyzed groups. There are many different preprocessing algorithms for summarizing Affymetrix data. The main goal of these methods is to remove effects of non-specific hybridization, and to optimally combine information from multiple probes annotated to the same transcript. The methods are benchmarked by comparison with reference methods, such as quantitative reverse-transcription PCR (qRT-PCR).

**Results:**

We present a comprehensive analysis of agreement between Affymetrix GeneChip and qRT-PCR results. We analyzed the influence of filtering by fraction Present calls introduced by J.N. McClintick and H.J. Edenberg (2006) and 2 mapping procedures: updated probe sets definitions proposed by Dai et al. (2005) and our "naive mapping" method. Because of evolution of genome sequence annotations since the time when microarrays were designed, we also studied the effect of the annotation release date. These comparisons were prepared for 6 popular preprocessing algorithms (MAS5, PLIER, RMA, GC-RMA, MBEI, and MBEImm) in the 2 above-mentioned types of studies. We used data sets from 6 independent biological experiments. As a measure of reproducibility of microarray and qRT-PCR values, we used linear and rank correlation coefficients.

**Conclusions:**

We show that filtering by fraction Present calls increased correlations for all 6 preprocessing algorithms. We observed the difference in performance of PM-MM and PM-only methods: using MM probes increased correlations in fold change studies, but PM-only methods proved to perform better in detection of differential expression. We recommend using GC-RMA for detection of differential expression and PLIER for estimation of fold change. The use of the more recent annotation improves the results in both types of studies, encouraging re-analysis of old data.

## Background

Affymetrix GeneChip microarrays (MA) are routinely used for genome-wide quantitative expression analysis. MA measure the expression of genes using probe sets. Probe sets are composed of multiple probes covering different regions of a particular transcript (Perfect-Match, PM), as well as probes designed to measure non-specific hybridization (Mismatch, MM).

Preprocessing algorithms evaluate the signals from probes and combine them to get an expression measure for each probe set. As was shown in [1-4], the choice of the preprocessing algorithm has a strong impact on MA analysis. *Irizarry et al*. [5] presented a comprehensive comparison of such algorithms and concluded that different preprocessing algorithms may suit different applications. In this study, we analyzed 6 popular algorithms: MAS5 [6], PLIER [7], RMA [8], GC-RMA [9], and 2 variants of MBEI algorithm [10,11], using either PM-only model (MBEI) or PM-MM model (MBEImm).

Quantitative reverse-transcription PCR (qRT-PCR) is another method to measure gene expression. This is a widely used diagnostic assay, but the measurements obtained with this technique are more costly and time consuming (per gene) than in MA analysis. In biological studies [12], as well as in analyses of preprocessing algorithms [13], qRT-PCR is widely used to validate MA results. As was shown in [3], the correlation coefficient between log-transformed (*log*_2_) microarray expression measurements and Δ*C*_*T *_values from qRT-PCR is a good measure of agreement.

Depending on the type, MA consist of several or tens of thousands probe sets, and traditional analysis of microarray data requires testing of all possible probe sets. However, multiple testing increases the number of false positives. For this reason, methods that eliminate unreliable measurements are helpful. One of them is filtering by fraction Present calls (*F*) which was proposed by *McClintick and Edenberg *[14] (for details see the Methods section). This method uses the detection call (Present/Absent/Marginal or P/A/M) originally designed to support the Affymetrix Microarray Suite 5 (MAS5), one of the first preprocessing algorithms. The P/A/M procedure uses both PM and MM probes to determine specificity of signal of each probe set. The filtering by fraction Present calls removes probe sets that had the fraction of Present calls below a given threshold in every sub-group of samples. The authors showed that *F *can be effectively applied even for data generated by another preprocessing algorithm. Recently, *Hackstadt and Hess *[15] compared different filtering methods on 3 biological data sets and pointed that *F *can increase the number of differentially expressed genes identified.

As was shown by *Okoniewski et al*. [16], there are many probe sets which are annotated ambiguously to several genes, so cross-hybridization signal might be seen. The authors demonstrated that such probe sets could hamper inference of gene interaction networks, and suggested the use of a comprehensive annotation of probe sets to genes. An example of such mapping are custom definition files packages (CDF packages denoted in our work as *D*), designed by *Dai et al*. [17]. Their redefinitions of probe sets are based on the latest genomic knowledge, and by regrouping probes and creating new probe sets, allow to limit analysis only to the gene-specific measurements. However, among the redefined probe sets the numbers of probes are not identical. Since such disparity may cause different quality of results, we implemented "naive mapping" method (*N*) which reduces the set of analyzed probe sets only to probe sets annotated to a single gene (for details see the Methods section). In our study of filtering/mapping effects, we compared 6 approaches: *A *- use of all probe sets without any filtering or mapping method, *F*, *D*, *N *as described above, and all their nontrivial combinations - *FD *and *FN*.

Both *D *and *N *mappings demand an annotation of probe sets to genes, and there are many different databases or probe set definitions for this purpose. Sandberg and Larsson [18] compared the accuracy in estimation of fold change computed with MA for 6 databases: NetAffx (original), ENSEMBL gene, ENSEMBL transcript, Entrez, RefSeq, and UniGene. Accuracy was defined as a slope after a linear regression between qRT-PCR and MA fold change for 16 genes. The authors observed the most significant improvement in accuracy when using the ENSEMBL gene and transcript databases (Figure 2 in [18]). Since qRT-PCR data used in our comparisons are labelled with the gene rather than the transcript names, we decided to use the ENSEMBL gene database.

Because of the evolution of genome sequence annotations, probe sets that originally were mapped uniquely to one gene may now be mapped to several genes. Therefore, to check the influence of this evolution of annotation, we decided to use 2 different versions of annotations: the *new *(ENSEMBL version 55 issued in Jul 2009 and custom CDF version 12 issued in Jul 2009) and the *old *(ENSEMBL version 49 issued in Mar 2008 and custom CDF version 11 issued in Nov 2008).

Typically, MA experiments are designed to profile the expression in two types of study: detection of differentially expressed genes [1,3,19] or estimation of fold change [13]. The difference of expression is usually measured by p-values of Welch's *t*-statistic that can be calculated as follows:

where  and  stand for means of log-transformed expressions in two analyzed groups,  and  are estimators of variances, and *n*_1 _and *n*_2 _denote the numbers of samples in each of the analyzed groups. In case of log-transform data fold change is simply computed as the difference between the means [13]:

In spite of the fact that *t *is a normalized *fc*, with regard to standard deviation, these two measurements have different motivations and lead to different results. Small p-values of Welch's *t*-test allow to point significantly differentially expressed genes, but possibly with small *fc*. Although possibly biologically relevant, such genes may be difficult to validate with qRT-PCR. On the other hand, scientists may be interested in changes of expression level between groups, measured by *fc *[13], but this statistic does not take into account a variability within groups. Thus, a large relative difference may be statistically insignificant. Moreover, there are approaches proposed to analyze sets of related genes (e.g. *Subramanian et al*. [20]) which benefit from the ranking of genes based on fold change regardless of significance of differential expression. Additionally, in the analysis of differentially expressed genes the ranking of calculated p-values or, equivalently, values of *t *is interesting, while in the analysis of fold change nominal values are interesting. Therefore, these two types of study should be treated differently. In our study, we used Spearman's correlation (rank correlation) to compare values of Welch's *t*-test from MA and qRT-PCR, and Pearson's correlation to compare the respective *fc *values.

Note that our analysis of agreement between detection of differential expression computed from qRT-PCR and MA results fits to the practice of preparing a list of genes with expected FDR below a given threshold. Indeed, a list of genes with expected *FDR *≤ *α *is a list with p-values not greater then some  dependent on *α *(this fact results from a characterization of FDR by q-values - for details see [21]). Hence, the more similar the lists of genes computed from MA and qRT-PCR results are, the higher a rank correlation between p-values is.

To sum up, we analyzed the influence of:

• type of MA study (fold change and differential expression);

• version of annotation of probe sets to genes (*new *and *old*);

• type of preprocessing algorithm (MAS5, PLIER, GC-RMA, RMA, MBEI, and MBEImm);

• type of filtering/mapping (*A*, *F*, *D*, *N*, *FD*, *FN*);

on the correlation between MA and qRT-PCR results.

## Results and Discussion

### Collected data sets

We collected publications describing both, the MA based gene expression profiling and validation performed on the same RNA samples with qRT-PCR experiments. Next, we asked authors to provide us with raw data (if not publicly available), i.e. microarray CEL files and tables of a threshold cycle (*C*_*T*_) from qRT-PCR. In this way, we obtained 6 data sets collected in different laboratories. The data sets are listed and abbreviated as follows:

• *NerdErd *- Samples from patients with non-erosive or erosive reflux disease [22]. We selected 2 groups which were well balanced in respect of sex and the batch effect.

• *Strain *- Data come from the study of effects of morphine administrations in 4 inbred mice strains [23]. We selected the contrast between 129P3/J and C57BL/6 strains after morphine, because it displays the greatest differences among 4 analyzed strains.

• *AgeWT *- Age difference (young/old) among wild-type mice. This is one of the most significant contrasts from an unbalanced 2x2 factorial design study [13] and was analyzed there in detail. We chose this contrast to facilitate comparison with the original study.

• *Lonely *- Samples of peripheral blood from human individuals differing in the degree of social interactions (high- and low-lonely) used in [12].

• *Tsc *- Brain specimens, consisting of 4 tumour samples from patients with tuberous sclerosis complex (Tsc) and 3 control samples. The data were performed in the Nencki Institute of Experimental Biology [24], and the MA can be obtained from ArrayExpress [25] (accession number E-MEXP-2351) while additional file 1 contains qRT-PCR results.

• *Maqc_CD *- In the MAQC project [26] gene expression levels were measured from 2 high-quality, commercially available RNA sample types *A *and *B *(Universal Human Reference RNA (UHRR) from Stratagene, Human Brain Reference RNA (HBRR) from Ambion). However, the authors noticed that in practical applications the expected differences between sample types were usually much smaller, and they suggested that mixtures *C *and *D *of the original samples were more realistic substitutes of biological samples (page 1157). We followed this suggestion.

For the *NerdErd *[22], *Strain *[23], *AgeWT *[12], and *Maqc_CD *[26] data sets, we analyzed only one contrast selected from the original multifactorial data. Using the data for all possible contrasts would upset the balance between the experiments in the final average towards the data sets with more groups. Our work, to our knowledge, is the first validation of preprocessing algorithms and filtering/mapping methods on independent data sets collected to address biological questions.

Table 1 presents more details about all described data sets. Let us notice that:

**Table 1:** Summary of experiments

Experiment			Microarrays	qRT-PCR
**name**	**ref**	**#samp**	**type**	**protocol**

*NerdErd*	[22]	38	hgu133a2	SybrGreen
*Strain*	[23]	36	mouse430_2	TaqMan
*AgeWT*	[13]	11	mgu74av2	TaqMan
*Lonely*	[12]	14	hgu133a	TaqMan
*Tsc*	[24]	7	hgu133plus2	SybrGreen
*Maqc_CD*	[26]	8	hgu133plus2	TaqMan

The first three columns contain general information about collected experiments: labels of the data sets used in our analysis (name), reference numbers (ref), and numbers of used samples (#samp). The fourth column describes a type of microarray used in each experiment: (type). The last column contains information about qRT-PCR protocol.

• The analyzed samples originate from different species and were hybridized on different MA types. This variety allows us to assume that the obtained average performance of different ways of MA data analysis is more representative for practical applications.

• Different qRT-PCR protocols were used to validate MA results in different experiments. However, *Arikawa et al*. [27] demonstrated that both SYBR Green and TaqMan delivered highly comparable results and both showed high agreement with MA data.

• The collected qRT-PCR data are labelled with gene rather than transcript names and we were not able to gather details of the primers used. Therefore, to compare MA and qRT-PCR results, we had to use annotation of probe sets to genes. In all variants of filtering/mapping except for *D *and *FD*, several different probe sets may be annotated to one gene. Therefore, it is necessary to transform expression measurements from probe set ensembles to genes. In this study we considered two such transformations: (i) assigning the expression measure from the best probe set (a probe set with the smallest p-value of the Welch's *t*-test) to a gene and (ii) averaging expression measurements over all probe sets annotated to the same gene. It turned out that choosing the best probe set led to consistently higher correlations between MA and qRT-PCR results (both *t *and *fc*), so we present only the results obtained with this method (for details see Methods section and additional file 2).

For all experiments and in each variant of analysis, a number of correlated genes depended on the version of annotations. Table 2 shows a number of genes correlated in each variant and experiment. In each cell, the first number stands for the new annotation, whereas the number in brackets stands for the old annotation.

**Table 2:** Number of correlated genes

	*NerdErd*	*Strain*	*AgeWT*	*Lonely*	*Tsc*	*Maqc_CD*
A	8 (8)	9 (9)	33 (35)	6 (6)	11 (10)	859 (876)
N	8 (7)	9 (9)	33 (31)	6 (6)	11 (10)	845 (793)
D	8 (8)	8 (8)	28 (33)	6 (6)	10 (10)	835 (838)
F	8 (8)	9 (9)	33 (35)	5 (5)	7 (6)	710 (731)
FN	8 (7)	9 (9)	33 (31)	5 (5)	7 (6)	697 (645)
FD	7 (7)	8 (8)	27 (32)	5 (5)	6 (6)	669 (669)

Number of correlated genes in each variant of filtering/mapping (rows) and a biological experiment (columns). In each cell, the first number stands for the *new *annotation, whereas the number in brackets stands for the *old *annotation.

### Detection of differentially expressed genes

For every gene validated with qRT-PCR, we calculated Welch's *t*-statistic, both from MA expression measurements and from Δ*C*_*T *_of qRT-PCR in a given experiment. The two resulting vectors of *t*-statistics (MA and Δ*C*_*T*_) were compared by calculating Spearman's correlation. For reasons of simplicity, since high Δ*C*_*T *_values correspond to low microarray expressions, we multiplied correlations by -1 in figures, and by -100 in tables.

**Figure 1:**
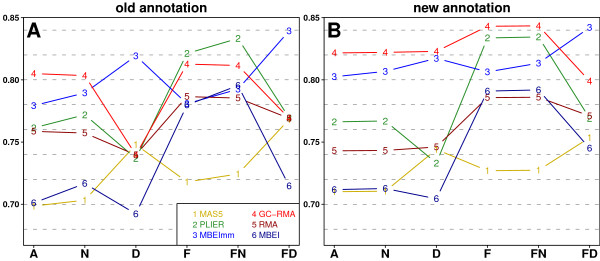
**Average Spearman's correlations between MA and qRT-PCR detection of differentially expressed genes**. Plots present average correlations between values of Welch's *t*-statistic calculated from MA and qRT-PCR measurements. Average values were calculated for each of the 6 preprocessing algorithms over the 6 data sets in each of the 6 filtering/mapping methods. Panel A shows results for the *old *annotation while panel B presents results for the *new *annotation. Curves are coloured and numbered to indicate the preprocessing algorithms.

Figure 1 shows average correlations for each of the 6 preprocessing algorithms over the 6 data sets in each of 6 filtering/mapping methods. Plot 1A and 1B present results for the *old *and *new *annotations, respectively. Overall, we observed that using the *new *annotation led to higher correlations, and the highest correlations were achieved in *F *and *FN*. For the *old *annotation a positive interaction between *N *and *F *was seen. However for the *new *annotation naive mapping had almost no influence, therefore, the correlations in *F *and *FN *were very similar. Effects of *D *strongly depended on preprocessing algorithms and, in comparison to *N*, did not show general improvement. Table 3 presents numerical values of the average correlations depicted on Figure 1 and their standard deviations (sd). We can see that GC-RMA, PLIER, and MBEImm outperform RMA, MAS5, and MBEI. Moreover, GC-RMA and PLIER achieved the highest correlations with qRT-PCR in the variants F and FN. On the other hand, MBEImm results are the most correlated with qRT-PCR in the variant FD, but sd of this method is significantly higher then sd of GC-RMA and PLIER.

**Table 3:** Average Spearman's correlations between MA and qRT-PCR detection of differentially expressed genes

		MAS5	PLIER	MBEImm	GC-RMA	RMA	MBEI
*new *annotation	*A*	71 ± 19	77 ± 9	80 ± 14	82 ± 9	74 ± 17	71 ± 15
	*N*	71 ± 19	77 ± 9	81 ± 14	82 ± 9	74 ± 17	71 ± 15
	*D*	74 ± 8	73 ± 15	82 ± 10	82 ± 8	75 ± 13	70 ± 13
	*F*	73 ± 20	83 ± 9	81 ± 13	84 ± 10	79 ± 19	79 ± 13
	*FN*	73 ± 20	83 ± 10	81 ± 14	84 ± 10	79 ± 19	79 ± 13
	*FD*	75 ± 15	77 ± 15	84 ± 13	80 ± 11	77 ± 16	74 ± 10

*old *annotation	*A*	70 ± 20	76 ± 11	78 ± 16	81 ± 12	76 ± 14	70 ± 15
	*N*	70 ± 21	77 ± 11	79 ± 15	80 ± 14	76 ± 15	72 ± 15
	*D*	75 ± 8	74 ± 14	82 ± 11	74 ± 14	74 ± 14	69 ± 15
	*F*	72 ± 22	82 ± 10	78 ± 15	81 ± 13	79 ± 14	78 ± 16
	*FN*	72 ± 22	83 ± 9	79 ± 14	81 ± 15	79 ± 15	80 ± 15
	*FD*	77 ± 13	77 ± 15	84 ± 14	77 ± 16	77 ± 16	71 ± 13

The table contains mean values and the corresponding standard deviations of Spearman's correlation coefficients calculated for 6 experiments. Correlations were computed between values of Welch's *t*-statistic calculated from MA and qRT-PCR measurements. Columns contain results for the 6 preprocessing algorithms, whereas rows contain results for the 6 filtering/mapping methods (negative signs are removed and values are multiplied by 100) after the *new *and the *old *annotations.

**Figure 2:**
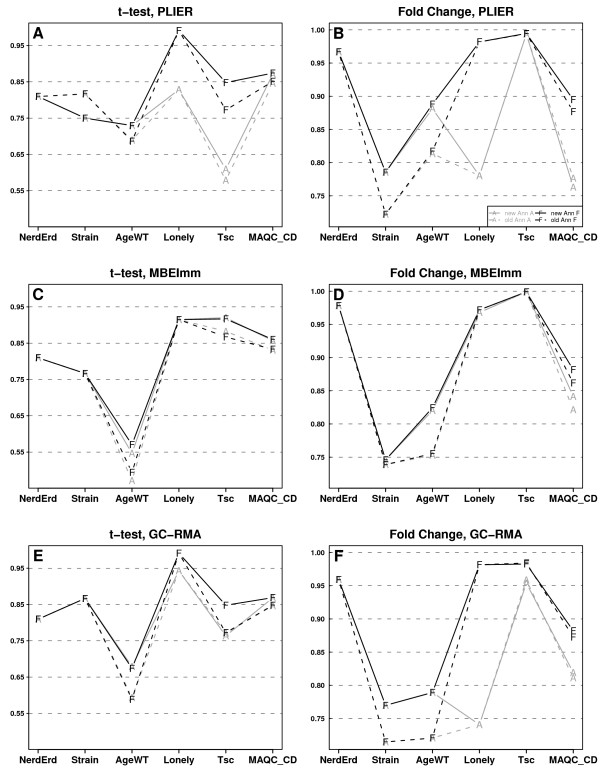
**Correlations of the most reproducible preprocessing algorithms**. Plots of correlations for 2 filtering/mapping methods, *A *and *F*, and both annotations in 2 applications (estimation of fold change and detection of differential expression), obtained for 3 preprocessing algorithms (PLIER, MBEImm, and GC-RMA). Colours denote filtering/mapping methods: *A *- grey, *F *- black while a line style stands for annotation: solid line - the *new *annotation, dashed line - the *old *annotation.

Figure 2 (parts A, C and E) shows correlations of 3 the best preprocessing algorithms for individual data sets in the variants *A *and *F *for both annotations. Let us notice that, for a given annotation, correlations in *F *are not lower than in *A*. Moreover, the highest correlations were achieved in *F *and the *new *annotation in all but one experiment (PLIER in *Strain *data).

### Estimation of the fold change

**Figure 3:**
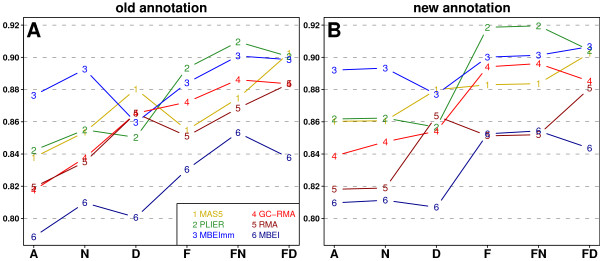
**Average Pearson's correlations between MA and qRT-PCR fold change**. Plots present average correlations between values of fold change calculated from MA and qRT-PCR measurements. Plot A shows results for the old annotation while plot B presents results for the *new *annotation. Curves are coloured and numbered to indicate the preprocessing algorithms.

Similarly as for detection of differential expression, we compared vectors of MA and qRT-PCR fold changes, but this time we used Pearson's correlations. Figure 3 presents results in a similar way to Figure 1. Overall, we can see that for each preprocessing algorithm and for both annotations correlations are higher than for the differential expression study. Again, the use of the *new *annotation generally leads to higher correlations. Moreover, for each preprocessing algorithm, the highest correlations were achieved in *FN *for the *old *annotation, and in *F *and *FN *for the *new *annotation. However, for the *new *annotation the influence of naive mapping was even smaller than in our differential expression study. The redefinitions *D *did not have equal influence on all preprocessing algorithms. For both annotations, *D *led to higher correlations, relatively to *N*, for GC-RMA, RMA, and MAS5, and to lower correlations for PLIER, MBEI, and MBEImm. Table 4 presents average correlations shown in Figure 3 and their standard deviations (sd). Like above, the highest correlations were achieved by PLIER in the variants F and FN. Moreover, the PLIER correlations again have the lowest sd. In general, PM-MM methods, outperformed PM-only methods regardless of annotations.

**Table 4:** Average Pearson's correlations between MA and qRT-PCR for estimation of the fold change

		MAS5	PLIER	MBEImm	GC-RMA	RMA	MBEI
*new *annotation	*A*	86 ± 11	86 ± 10	89 ± 10	84 ± 9	82 ± 14	81 ± 13
	*N*	86 ± 11	86 ± 10	89 ± 10	85 ± 10	82 ± 14	81 ± 13
	*D*	88 ± 9	86 ± 10	88 ± 10	85 ± 7	86 ± 9	81 ± 14
	*F*	88 ± 11	92 ± 8	90 ± 10	89 ± 10	85 ± 15	85 ± 14
	*FN*	88 ± 11	92 ± 8	90 ± 10	90 ± 10	85 ± 15	85 ± 14
	*FD*	90 ± 9	90 ± 9	91 ± 11	89 ± 6	88 ± 9	84 ± 15

*old *annotation	*A*	84 ± 13	84 ± 11	88 ± 12	82 ± 11	82 ± 13	79 ± 15
	*N*	85 ± 13	86 ± 11	89 ± 10	84 ± 11	84 ± 11	81 ± 13
	*D*	88 ± 9	85 ± 10	86 ± 11	87 ± 9	87 ± 9	80 ± 14
	*F*	85 ± 13	89 ± 11	88 ± 12	87 ± 13	85 ± 14	83 ± 17
	*FN*	87 ± 13	91 ± 10	90 ± 10	89 ± 11	87 ± 12	85 ± 14
	*FD*	90 ± 9	90 ± 9	90 ± 12	88 ± 9	88 ± 9	84 ± 16

The table contains mean values and the corresponding standard deviations of Pearson's correlation coefficients. Correlations were computed between fold changes calculated from MA and qRT-PCR measurements. Columns contain results for the 6 preprocessing algorithms, whereas rows contain results for the 6 filtering/mapping methods (negative signs are removed and values are multiplied by 100) after the *new *and the *old *annotations.

Figure 2 (parts B, D, and F) presents correlations of 3 the best preprocessing algorithms for each experiment in the variants *A *and *F*, for both annotations. As before, correlations in *F *were not lower than in *A*, and the highest correlations were achieved in *F *and the *new *annotation.

### Unreliability of results for MBEImm

Although MBEImm leads to one of 2 the best results, both for detection of differentially expressed genes and for estimation of the fold change, there are some reasons that undermine this method. Raw MBEImm values may be negative and cannot be log-transformed (such observations are omitted by default). If this occurs for the best probe sets (see the Methods section), then the values of *t*-statistic or fold change are computed using lower number of observations than in case of other preprocessing algorithms or qRT-PCR. For example, Figure (2B, D and 2F) shows that MBEImm clearly outperformed GC-RMA and PLIER in A and N filtering/mapping variants for *Lonely *data. This result implies a similar order of preprocessing algorithms as in Figure 3, but, as we checked, the values of *fc *and t for MBEImm were computed using a lower number of observations than in case of the other preprocessing algorithm. Thus, correlation of MBEImm, and qRT-PCR should not be compared with other preprocessing algorithms. Moreover, in Tables 3 and 4 we show that MBEImm results are less stable (greater sd) than for PLIER or GC-RMA.

### Principal Component Analysis of preprocessing algorithms

**Figure 4:**
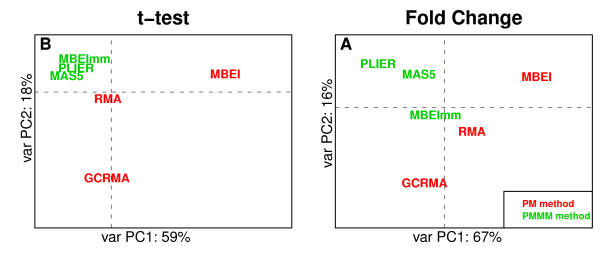
**Principal Component Analysis of preprocessing algorithms in variant A**. Plots present Principal Component Analysis of preprocessing algorithms on sample types A and B from MAQC study. Algorithms using the MM probes are marked green while the PM-only algorithms are marked red. (A) PCA of values of Welch's *t*-statistic from microarray and qRT-PCR detection of differentially expressed genes. (B) PCA of values of fold change between groups. In both cases the *new *annotation was used. Axes are labelled with percentage of total variability explained by PC1 and PC2. It should be noted that in this technical comparison we used samples of original RNA instead of mixtures C and D.

Comparison of Table 3 and Table 4 suggests that there is a difference in performance of PM-MM and PM-only methods. In the FN variant of filtering/mapping (generally the best one) using MM probes increased correlation in the fold change studies, but PM-only methods performed better in the detection of significant expression. We confirmed the difference in performance of PM-MM and PM-only preprocessing algorithms in Principal Component Analysis on MAQC data. We used A and B sample types, instead of C and D because we wanted to obtain the maximal biological diversity and to analyze only technical differences. We performed PCA on values of *t*-test and fold change separately using only genes that were validated with qRT-PCR. Then, we plotted the preprocessing algorithms on the plane of the first (PC1) and the second (PC2) principal directions. Figure 4 presents both results.

The relevance of PC1 and PC2 measured with a sum of relative variances of first two PC amounts to 77% for detection of differentially expressed genes, and to 83% for estimation of fold change. Discrimination between PM-only PM-MM methods is well noticeable, especially in Figures 4A, where PC1 separates PM-only from PM-MM methods. Additionally, in both pictures, the PM-MM algorithms are placed more closely to each other. In both cases, MBEI and MBEImm algorithms are separated by the first principal component.

## Conclusions

This paper presents a comparison of preprocessing algorithms and filtering/mapping variants for analysis of microarray data. Based on 6 independent biological data sets, we studied correlations between MA and qRT-PCR expression measurements in 2 studies (detection of differentially expressed genes and estimation of fold change) using 2 annotations (*new *and *old*). We showed that filtering by fraction Present calls increased correlations for all 6 preprocessing algorithms. We observed the difference in performance of PM-MM and PM-only methods: using MM probes increased correlations in the fold change studies, but PM-only methods performed better in the detection of significant expression. For detection of differentially expressed genes, we recommend using GC-RMA, and for estimation of fold change - PLIER algorithm. The use of the more recent annotation improves results in both types of studies, encouraging re-analysis of old data.

## Methods

### RNA assays

As the *NerdErd*, *Strain*, *AgeWT*, and *Lonely *data were taken from already published papers, we describe only the generation of the *Tsc *data. Total RNA was prepared by Tri-Reagent (Sigma) extraction from snap-frozen tissues and cleaned up using RNeasy Mini Kit according to the manufacturer's protocol (Qiagen). The quality and quantity of RNA were verified using the Agilent bioanalyzer (Agilent Technologies). All the microarray preparation procedures were done according to recommendations of Affymetrix (Santa Clara, CA) using 5 *μ*g of total RNA as a template. Fragmented cRNA was hybridized first to a control microarray (Test3) and then, after sample quality evaluation, to the HG-U133 Plus 2.0 arrays (Affymetrix). Samples hybridization was done in the Department of Nuclear Medicine and Endocrine Oncology, Maria Sklodowska-Curie Memorial Cancer Center and Institute of Oncology, Gliwice, Poland. The cDNAs were synthesized by extension of *oligo*(*dT*)_15 _primers with 200 units of M-MLV reverse transcriptase (Sigma) in a mixture containing 1 *μ*g of total RNA in 20 *μ*l. Real-time PCR analysis was performed in duplicate using the 7500 Real Time PCR System (Applied Biosystems) on cDNA equivalent to 10 ng RNA in 20 *μ*l reaction volume containing 1× SYBR Green PCR master mix (Applied Biosystems) and the primer sets QuantiTect Primer Assays (200) (Qiagen). 18SrRNA was used as an internal control with primers designed by the Primer Express Software (Applied Biosystems).

### Filtering by fraction Present calls

P/A/M detection algorithm was originally designed for a single array analysis to verify if a particular probe set measured specific or non-specific hybridization signal. This algorithm is based on Wilcoxon signed rank test and was introduced by Affymetrix [6]. Each analyzed probe set gets one of three possible labels depending on signals of PM and MM probes. Label P (Present) means that the signal is specific, while label A (Absent) stands for the lack of specific signal. The third label, M (Marginal), means that specificity is not clear.

We implemented one variant of filtering by fraction Present calls proposed in [14]. We removed probe sets which got less than 25% percent of calls P in the analyzed experiments. Only for the *Tsc*, we decided to raise the threshold to 50%, because of a small number of samples. A similar approach, but without using information about the treatment assignments, was used in [15].

### Naive mapping

Naive mapping consists in exclusion of all probe sets that are annotated to more than one gene (one-to-many). To do so, we used annotations of probe sets to genes provided in the Ensembl database (identifiers with ENSG prefix in human or ENSMUSG prefix in mice) [28].

### Calculation of gene expression from probe sets annotated to the same gene

In probe sets to gene annotation, it may be observed that (i) only one probe set is annotated to a given gene or (ii) several probe sets are annotated to the same gene (annotation of one probe set to more that one gene is a filtering/mapping problem). In the first case, we simply used the received signal and in the second case, we used the best probe set (probe sets with the smaller p-values of *t*-test among all probe sets annotated to the same gene) to quantify gene *t*-test or fold change. For all analyzed data sets, there were ensembles of probe sets annotated to the same gene irrespective of the filtering/mapping variants (besides *FD *and *D*).

We also tried to evaluate gene-specific values without consideration of the treatment assignments. For each microarray, we computed an arithmetic mean as well as a Tukey biweight. Both ways of averaging led to similar results and were outperformed by the "best probe set" method. In Additional file 2, we present results only for an arithmetic mean, because it is easier and faster to compute it than a Tukey biweight function.

### Preprocessing of microarray data

All calculations were performed in R statistical environment [29] and relevant Bioconductor software [30]. The *mas*5( ), *rma*( ), and *gcrma*( ) functions were used with default parameters. To apply MBEI and MBEImm, we used *expresso*( ) function according to description in [31]. To apply PLIER, we used *justPLIER*( ) function from plier package with a normalize parameter set on TRUE according to recommendation in [7]. Before further analysis, we log-transformed the results of MAS5, MBEImm, and MBEI. The example of used R code is introduced in Additional file 3.

## Authors' contributions

JM and PP designed the study and wrote R scripts. MD provided biological expertise to the microarrays technology and the Ensembl annotation. MT prepared all RNA and performed the qRT-PCR experiment for the Tsc data set. JM performed the statistical analysis and drafted the manuscript. JM, PP, MT and MD wrote the manuscript. All authors read and approved the final manuscript.

## Supplementary Material

Additional file 1 **This file contains qRT-PCR results of Tsc experiment**. Click here for file

Additional file 2 This is a PDF document showing average correlations between 6 preprocessing algorithms and qRT-PCR in 2 studies (fold change, *t*-test) and 2 annotations (*new*, *old*). We present results obtained with the best probe set transformation, as well as with the mean probe set transformation. The tables numbered even correspond to Tables 3 and 4. Click here for file

Additional file 3 **This document contains an R code used in our analysis**. Click here for file
