# Supplementary material for: Probe set filtering increases correlation between Affymetrix GeneChip and qRT-PCR expression measurements
Source: BMC Bioinformatics. 2010 Feb 24;11:104. doi: 10.1186/1471-2105-11-104 (PMC2841208; doi:10.1186/1471-2105-11-104)
Supplement: Additional file 2 — This is a PDF document showing average correlations between 6 preprocessing algorithms and qRT-PCR in 2 studies (fold change, t-test) and 2 annotations (new, old). We present results obtained with the best probe set transformation, as well as with the mean probe set transformation. The tables numbered even correspond to Tables 3 and 4. [file 1471-2105-11-104-S2.PDF]

New Annotation

Fold Change

mean

	MAS5	PLIER	MBEImm	GC-RMA	RMA	MBEI
A	87±11	85±10	87±8	86±8	86±9	82±12
N	87±11	83±13	87±8	86±8	86±8	82±12
D	88±9	86±10	88±10	85±7	86±9	81±14
F	89±10	92±7	90±8	90±7	89±8	86±13
FN	89±10	89±7	90±8	90±8	89±8	86±13
FD	90±9	90±9	91±11	89±6	88±9	84±15

best

	MAS5	PLIER	MBEImm	GC-RMA	RMA	MBEI
A	86±11	86±10	89±10	84±9	82±14	81±13
N	86±11	86±10	89±10	85±10	82±14	81±13
D	88±9	86±10	88±10	85±7	86±9	81±14
F	88±11	92±8	90±10	89±10	85±15	85±14
FN	88±11	92±8	90±10	90±10	85±15	85±14
FD	90±9	90±9	91±11	89±6	88±9	84±15

t–test

mean

	MAS5	PLIER	MBEImm	GC-RMA	RMA	MBEI
A	68±15	73±11	75±16	80±10	75±16	71±21
N	67±15	72±12	75±16	80±11	75±16	71±22
D	74±8	73±15	82±10	82±8	75±13	70±13
F	69±20	78±14	77±13	79±14	80±16	75±17
FN	69±20	78±14	77±13	79±14	80±16	75±17
FD	75±15	77±15	84±13	80±11	77±16	74±10

best

	MAS5	PLIER	MBEImm	GC-RMA	RMA	MBEI
A	71±19	77±9	80±14	82±9	74±17	71±15
N	71±19	77±9	81±14	82±9	74±17	71±15
D	74±8	73±15	82±10	82±8	75±13	70±13
F	73±20	83±9	81±13	84±10	79±19	79±13
FN	73±20	83±10	81±14	84±10	79±19	79±13
FD	75±15	77±15	84±13	80±11	77±16	74±10

Old Annotation

Fold Change

mean

	MAS5	PLIER	MBEImm	GC-RMA	RMA	MBEI
A	86±10	84±10	85±9	84±10	84±11	80±15
N	88±10	83±13	87±8	85±9	86±9	82±12
D	88±9	85±10	86±11	87±9	87±9	80±14
F	88±11	90±10	88±10	88±11	87±12	83±16
FN	89±11	89±9	90±9	90±10	88±10	85±14
FD	90±9	90±9	90±12	88±9	88±9	84±16

best

	MAS5	PLIER	MBEImm	GC-RMA	RMA	MBEI
A	84±13	84±11	88±12	82±11	82±13	79±15
N	85±13	86±11	89±10	84±11	84±11	81±13
D	88±9	85±10	86±11	87±9	87±9	80±14
F	85±13	89±11	88±12	87±13	85±14	83±17
FN	87±13	91±10	90±10	89±11	87±12	85±14
FD	90±9	90±9	90±12	88±9	88±9	84±16

t–test

mean

	MAS5	PLIER	MBEImm	GC-RMA	RMA	MBEI
A	68±14	72±14	73±17	80±12	75±17	72±21
N	68±15	72±14	74±17	79±13	75±17	73±21
D	75±8	74±14	82±11	74±14	74±14	69±15
F	72±20	76±17	75±15	77±18	79±19	75±17
FN	72±21	77±17	76±14	77±19	79±19	77±16
FD	77±13	77±15	84±14	77±16	77±16	71±13

best

	MAS5	PLIER	MBEImm	GC-RMA	RMA	MBEI
A	70±20	76±11	78±16	81±12	76±14	70±15
N	70±21	77±11	79±15	80±14	76±15	72±15
D	75±8	74±14	82±11	74±14	74±14	69±15
F	72±22	82±10	78±15	81±13	79±14	78±16
FN	72±22	83±9	79±14	81±15	79±15	80±15
FD	77±13	77±15	84±14	77±16	77±16	71±13
